# The potential effect of gut microbiota on the secretion of selected cytokines by human cells in vitro

**DOI:** 10.1038/s41598-025-01581-3

**Published:** 2025-05-19

**Authors:** Paulina Natalia Kopa-Stojak, Paulina Kleniewska, Arkadiusz Hoffmann, Rafał Pawliczak

**Affiliations:** Department of Immunopathology, Faculty of Medicine, Medical University of Lodz, Zeligowskiego 7/9, (bldg 2 Rm 177), 90-752 Lodz, Poland

**Keywords:** Asthma, Intestinal microflora, Airway inflammation, Immunology, Medical research, Pathogenesis

## Abstract

Colonization of the gut early in life plays a significant role in guiding the development of the immune system. The effect of individual intestinal bacterial strains on the asthma development is the subject of numerous scientific studies. The aim of the study was to determine the role and the potential mechanism of action of intestinal microflora on secretion of cytokines and potential predisposition to asthma development. The effect of *Parabacteroides distasonis (PD)*,* Bacteroides vulgatus (BV) Clostridium perfringens (CP)* and *Ruminococcus albus (RA)* lysates on the secretion of IL-4, IL-5, IL-8 (CXCL8) and IL-13 in human peripheral blood mononuclear cells, monocytes and HT-29 cells has been analyzed by enzyme-linked immunosorbent assays. RA and PD 400 µg lysates significantly increased secretion of IL-5 by PBMC compared to control (*p* < 0.05). In addition, BV, CP, PD and RA 100 µg lysates significantly increased IL-8 secretion by PBMC, as well as BV, PD and RA 100 µg lysates by monocytes compared to control (*p* < 0.05). Moreover, PD 100 µg and 400 µg lysates significantly increased secretion of IL-8 by HT-29 cells compared to BV 100 µg and 400 µg lysates (*p* < 0.05). CP, BV, PD and RA 100 µg lysates significantly lowered IL-13 secretion by PBMC vs. control (*p* < 0.001). For a better understanding of the mechanisms of action of gut microflora and their impact on parameters important in asthma, complex studies which compare the asthma and control samples should be carried out in the future.

## Introduction

Research on the relationship of the human microbiome with the incidence of asthma focuses mainly on its significant role in the development of the immune system in infancy^1–3^. Researches show an inverse correlation between the incidence of asthma and the amount of microorganisms in children’s environment^4^. The excessive use of antibiotics or increasingly sterile living conditions are indicated as reasons for a growing number of cases of asthma, which in turn contribute to the reduced exposure to antigens of microorganisms critical for the proper development of the immune system. This set of external factors related to the development of asthma is called “hygiene hypothesis”^5–7^. The loss of individual microflora species may cause rapid changes in the “hierarchy” of bacteria in the digestive system, to which our immune system may not be able to adapt, and consequently its regulation is disturbed^8^.

Recently, numerous studies on animal and human models indicate that colonization of the gut early in life plays a significant role in guiding the development of the immune system. Interference with this normal process can increase the likelihood of developing atopic disorders such as asthma. Scientists point to the first six months of a child’s life as “critical immune window” during which any change in the composition of the intestinal microflora may affect the subsequent development of the immune system, the emergence of allergic diseases and asthma^9^. The initial composition of intestinal bacterial flora of infants is treated as a key factor regulating the development of atopic diseases^6^.

According to the aforementioned hygienic hypothesis, intestinal microflora may act as an important regulator of the Th1 and Th2 immune balance, which is important in asthma and allergic diseases. In addition, it plays a large role in maintaining oral immune tolerance by inhibiting potential lymphocyte reactivity. The *Lactobacillus rhamnosus* GG and Streptococcus pyogenes (group A streptococci) strains activate the production of Th1 cytokines by NF-ĸB and STAT-dependent signaling pathways in macrophages^10^. Moreover, in vitro studies using peripheral blood mononuclear cells obtained from adult patients with pollen allergy showed that *Bacteroides fragilis* compared to *Bifidobacterium* strains induce more Th2 cells, which produce cytokines such as IL-4, IL-5 and IL-13 which stimulate B lymphocytes to produce IgE. At the same time, the production of Th1 cytokines is inhibited^11^.

Among the available publications that analyze the involvement of bacteria in the formation and development of asthma, it appears that the types most often associated with its advancement are *Bacteroides*, *Clostridium*, *Staphylococcus*, and *Enterobacteriaceae*. On the other hand, there are *Bifidobacterium* and *Lactobacillus*, which show protective effects^12^. Arrieta MC et al. research confirms earlier findings of dysbiosis in the first 100 days of a newborn’s life as a factor of increasing the risk of asthma later in life. However, in their opinion, instead of a total change in the composition of the intestinal flora, only 4 types of bacteria are crucial, including: *Faecalibacterium*, *Lachnospira*, *Veillonella*, and *Rothia*. Furthermore, they suggest that only changes very early in life could have any effect on the later development of asthma. In addition, *Faecalibacterium*, *Lachnospira*, *Veillonella*, and *Rothia* introduced into the digestive system of mice significantly alleviated the inflammation of the respiratory tract of their adult offspring compared with control group mice^13^. A similar study published by Fujimura KE et al. linked the presence of *Bifidobacterium*, *Akkermansia*, and *Faecalibacterium* in stool samples of one-month-old infants with a three-fold higher risk of asthma at the age of four^14^. Another study shown increased number of *Bacteroides* and reduced number of *Ruminococcaceae* in adult asthma patients compared to healthy controls. Moreover, asthma patients were characterized by an increased number of *Bacteroides* (including *Bacteroides vulgatus*), *Parabacteroides* (including *Parabacteroides distasonis*) or *Clostridium saccharolyticum*^15^. Huang K. et al. showed that intestinal *Clostridium perfringens* colonization is correlated with cow’s milk protein allergy (CMPA) and food allergy in infants^16^. Despite some promising examples, there is still no reliable epidemiological research taking into account the importance of intestinal microflora in the development of bronchial asthma. The aim of the study is to determine the role and potential mechanism of action of intestinal microflora on secretion of cytokines and potential predisposition to asthma development.

## Materials and methods

### Intestinal microflora strains

This research study was preceded by a pilot study, where significant differences in gut microbiota species between asthma patients and healthy controls were identified by next generation sequencing (NGS)^15^. Based on the results of such study, intestinal microflora strains: *Parabacteroides distasonis*, *Bacteroides vulgatus*,* Clostridium perfringens* and *Ruminococcus albus* have been chosen. *Parabacteroides distasonis* (ATCC 8503), *Bacteroides vulgatus* (ATCC 8482), *Clostridium perfringens* (ATCC 13124) and *Ruminococcus albus* (ATCC 27210) were obtained with the LGC Standards (Teddington, UK). *Parabacteroides distansonis* was harvested in ATCC 1490 Modified Chopped Meat medium, *Bacteroides vulgatus* was cultivated in ATCC 2107 Modified Reinforced Clostridial Medium, *Ruminococcus albus* was cultivated in ATCC 158 RGCA Medium and *Clostridium perfringens* was harvested in ATCC 2107 Modified Reinforced Clostridial medium (LGC Standards, Teddington, UK). Under anaerobic conditions, thawed vials with bacterial species were aseptically transferred to 5–6 mL tubes containing pre-reduced media and cultivated in 37^o^C for 24–48 h in a chamber designed for cultivating anaerobic bacteria strains (candle lit jar). The bacterial culture was transferred to fresh broth tubes every 24 to 48 h. ATCC 260 Trypticase soy agar with 5% sheep blood was used as a selection medium. One agar plate (for each bacteria strain) was incubate aerobically at 37 °C to check for contamination.

### Human cell lines

This research study based on commercially purchased cell lines. Human peripheral blood mononuclear cells (PBMC) were obtained from SigmaAldrich (Saint Louis, MO, USA), human HT-29 cells were obtained from LGC Standards (Teddington, UK). Human monocytes were isolated from PBMC. PBMC was cultivated in Mononuclear Cell Medium (SigmaAldrich, Saint Louis, MO, USA) in T75 flasks in standard conditions (37^o^C, 5% CO_2_, 90% humidity). To isolation of human monocytes, PBMC were incubated in RPMI-1640 medium without 10% FBS (SigmaAldrich, Saint Louis, MO, USA) in T75 flasks for 2 h in standard conditions, which allowed the monocytes to adhere to the surface of the culture flask (temporary adhesion in order to separate monocytes from other cells). Remaining cells were removed from the culture by washing three times in warm RPMI-1640 medium (SigmaAldrich, Saint Louis, MO, USA). Then isolated monocytes were harvested in RPMI-1640 medium (SigmaAldrich, Saint Louis, MO, USA) supplemented with 10% FBS (SigmaAldrich, Saint Louis, MO, USA), which reversed such temporary adhesion of monocytes to the surface of cell culture flasks and allow to normal harvesting of floating cells in the standard conditions (37^o^C, 5% CO_2_, 90% humidity). Human HT-29 cell line was harvested in McCoy’s 5 A medium (ATCC 30–2007) and fetal bovine serum (ATCC, 500 mL) in T75 flasks in standard conditions (37^o^C, 5% CO_2_, 90% humidity). Media were supplemented with an antibiotic mixture (SigmaAldrich, #P4333, Penicilin/Streptomycin; Lonza #195266). The authors checked the NCBI database for misidentification and contamination of human cell lines. Ethical approval was not required for this project. All methods were carried out in accordance with good laboratory practice and scientific requirements necessary for designing, conducting, collecting and analyzing of the research data.

### Preparation of bacterial lysates by ultrasonic disintegrator

*Parabacteroides distansonis*,* Bacteroides vulgatus*, *Clostridium perfringens*, *Ruminococcus albus* cultures were transferred to 2 mL eppendorfs and then centrifuged (5 min, 10000 rpm, 4^o^C). The bacterial cell pellets were then suspended in 1 mL of distilled water and disintegrated using an ultrasonic disintegrator. Then samples were centrifuged (5 min, 10000 rpm, 4^o^C) and the supernatants were transferred to new collection tubes and stored at −20^o^C.

### Stimulation of human peripheral blood mononuclear cells, monocytes and HT-29 cells by bacterial lysates

Human peripheral blood mononuclear cells were seeded in a 6-well plate at 2 × 10^6^ cells/well, human monocytes and human HT-29 at 0.5 × 10^6^ cells/well. Each cell line was stimulated with 100 µg and 400 µg of bacterial lysates (lysates concentration: 1.218 mg/ml RA, 1.68 mg/ml BV, 1.657 mg/ml CP and 1.687 mg/ml PD for PBMC and monocytes; 1.207 mg/ml RA, 1.288 mg/ml BV, 1.609 mg/ml CP and 1.397 mg/ml PD for HT-29 cells) for 24 h^17^. The cells incubated with fresh culture medium were a negative control, and with 25 µg/ml dexamethasone were positive control. After 24 h of incubation, the samples were centrifuged (5 min, 1500 rpm, 4^o^C) and supernatants were transferred to new collection tubes and stored at −20^o^C.

### Analysis of selected cytokines secretion by enzyme-linked immunosorbent assay

For analysis of IL-4, IL-5, IL-8 and IL-13 secretion by peripheral blood mononuclear cells, monocytes and HT-29 cells after 24 h incubation with bacterial lysates, enzyme-linked immunosorbent assays were performed according to the SigmaAldrich (Saint Louis, MO, USA) manufacturer’s instruction (IL-4 cat. no: RAB0298 (standard curve range: 3.13–200 pg/ml), IL-5 cat. no. RAB0303 (standard curve range: 2.74–2000 pg/ml), IL-8 (CXCL8) cat. no. RAB0319 (standard curve range: 0.8–600 pg/ml), IL-13 cat. no. RAB0256 (standard curve range: 0.16–40 pg/ml). 100 µL of standards and each sample in at least two repeats were added into 96-well ELISA plates and incubated in room temperature for 2.5 h with gentle shaking. Then, the solution was discarded and washed (4 times) with wash solution (300 µL/well). After the last wash, 100 µL of detection antibody was added into each well and incubated for 1 h at room temperature with gentle shaking. Next, washing step was repeated and 100 µL of streptavidin solution was added into each well for 45 min incubation (room temperature, gentle shaking). After this time the washing step was repeated as previously and 100 µL of substrate reagent was added to each well for 30 min (room temperature, in the dark, gentle shaking). Finally, 50 µL of stop solution was added and absorbance at 450 nm was read on the microplate reader.

### Statistical analysis

The final results are a mean of three independent repeats of human cells stimulation by bacterial lysates. The values are presented as mean ± SEM. The statistical analysis was done by ANOVA test followed by Tukey’s test and Dunnett’s method (comparison of samples to control and comparison between samples). The *p* < 0.05 was considered statistically significant.

## Results

### Parabacteroides distasonis, *Bacteroides vulgatus*, *Clostridium perfringens*, *Ruminococcus albus* effect on IL-4 secretion by PBMC

**Fig. 1:**
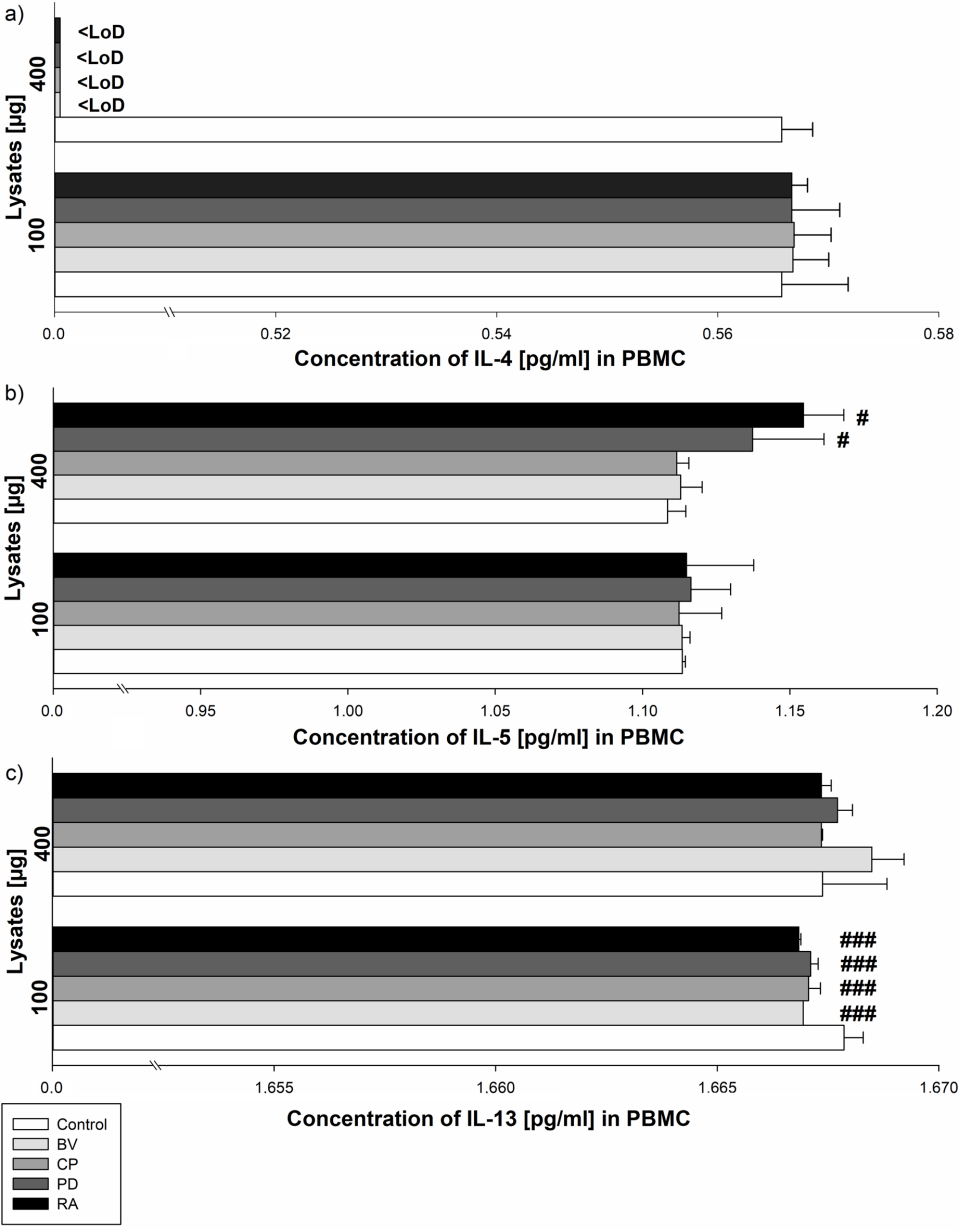
The effect of bacterial lysates (100 µg and 400 µg lysates) on IL-4 (a), IL-5 (b) and IL-13 (c) secretion by human peripheral blood mononuclear cells (PBMC). Human peripheral blood mononuclear cells were seeded in a 6-well plate at 2 × 10^6^ cells/well and stimulated with 100–400 µg of bacterial lysates for 24 h. The concentration of IL-4, IL-5 and IL-13 was measured by ELISA assay. The final results are the mean of three independent repeats of human cells stimulation by bacterial lysates. The values are presented as mean ± S.E.M; Tukey’s Test: # *p* < 0.05 vs. control group; ### *p* < 0.001 vs. control group. BV - *Bacteroides vulgatus*, CP - *Clostridium perfringens*, PD - *Parabacteroides distasonis*, RA - *Ruminococcus albus*; LoD - limit of detection.

Control and all 100 µg bacteria lysates were similar with respect to IL-4 secretion by PBMC. In addition, there were no significant differences in IL-4 secretion by PBMC between the bacteria strains. Furthermore, secretion of IL-4 by PBMC induced by 400 µg of lysates was under assay detection limit (LoD) (for each of three repeats) (Fig. 1a).

### Parabacteroides distasonis, *Bacteroides vulgatus*, *Clostridium perfringens*, *Ruminococcus albus* effect on IL-5 secretion by PBMC

Control and *Parabacteroides distasonis*, *Ruminococcus albus*,* Bacteroides vulgatus* and *Clostridium perfringens* 100 µg lysates were similar in respect to IL-5 secretion by PBMC. In addition, there were no significant differences in IL-5 secretion by PBMC between the bacteria strains. Furthermore, *Ruminococcus albus* and *Parabacteroides distasonis* lysates at a dose of 400 µg significantly increased IL-5 secretion by PBMC (1.15 ± 0.01 pg/ml for RA and 1.14 ± 0.02 pg/ml for PD) compared to control (1.11 ± 0.001 pg/ml; *p* < 0.05). All other 400 µg lysates and control were similar with respect to the secretion of IL-5 by PBMC (Fig. 1b).

### Parabacteroides distasonis, *Bacteroides vulgatus*, *Clostridium perfringens*, *Ruminococcus albus* effect on IL-13 secretion by PBMC

*Bacteroides vulgatus*,* Clostridium perfringens*,* Parabacteroides distasonis* and *Ruminococcus albus* 100 µg lysates significantly lowered IL-13 secretion by PBMC (1.67 ± 0.001 pg/ml for BV, 1.67 ± 0.001 pg/ml for CP, 1.67 ± 0.001 pg/ml for PD and 1.67 ± 0.001 pg/ml for RA) compared to control (1.68 ± 0.001 pg/ml; *p* < 0.001). Moreover, *Bacteroides vulgatus*,* Parabacteroides distasonis* 400 µg lysates and control were similar with respect to the secretion of IL-13 by PBMC. Finally, there were no significant differences in IL-13 secretion by PBMC between the bacteria strains (Fig. 1c).

### *Parabacteroides distasonis*, *Bacteroides vulgatus*, *Clostridium perfringens*, *Ruminococcus albus* effect on IL-8 secretion by PBMC, monocytes and HT-29 cells

**Fig. 2:**
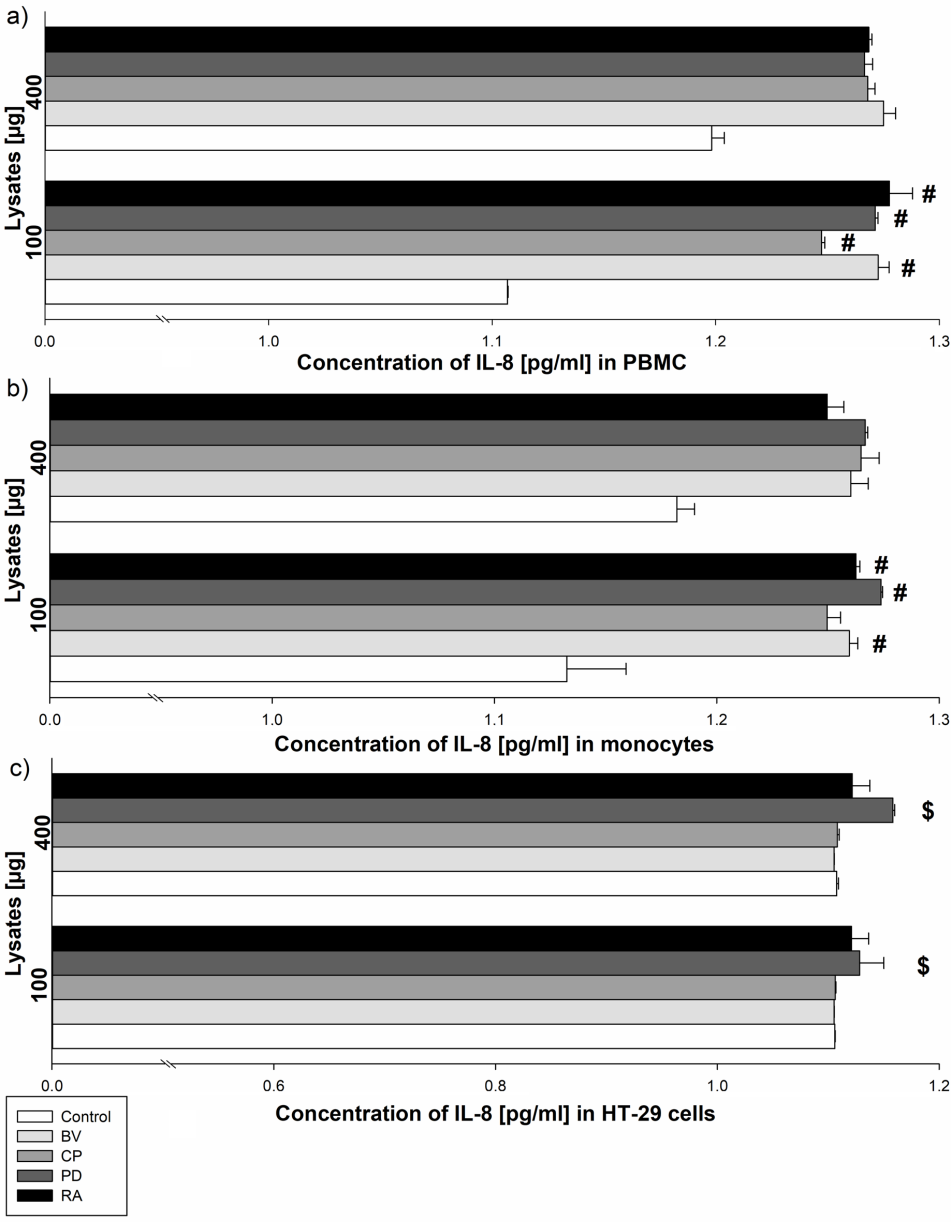
The effect of bacterial lysates (100 µg and 400 µg lysates) on IL-8 secretion by human peripheral blood mononuclear cells (PBMC) (**a**), human monocytes (**b**) and human intestinal epithelial cells (HT-29) (**c**). Human peripheral blood mononuclear cells were seeded in a 6-well plate at 2 × 10^6^ cells/well, human monocytes and human HT-29 at 0.5 × 10^6^ cells/well and stimulated with 100–400 µg of bacterial lysates for 24 h. The concentration of IL-8 was measured by ELISA assay. The final results are the mean of three independent repeats of human cells stimulation by bacterial lysates. The values are presented as mean ± S.E.M; Tukey’s Test: # *p* < 0.05 vs. control group; $ *p* < 0.05 vs. BV; BV - *Bacteroides vulgatus*, CP - *Clostridium perfringens*, PD - *Parabacteroides distasonis*, RA - *Ruminococcus albus.*

*Bacteroides vulgatus*,* Clostridium perfringens*,* Parabacteroides distasonis* and *Ruminococcus albus* 100 µg lysates significantly increased IL-8 secretion by PBMC (1.27 ± 0.005 pg/ml for BV; 1.25 ± 0.01 pg/ml for CP; 1.27 ± 0.001 pg/ml for PD and 1.28 ± 0.02 pg/ml for RA) compared to control (1.11 ± 0.003 pg/ml; *p* < 0.05). In addition, *Bacteroides vulgatus*,* Parabacteroides distasonis* and *Ruminococcus albus* 100 µg lysates significantly increased IL-8 secretion by monocytes (1.26 ± 0.003 pg/ml for BV; 1.27 ± 0.01 pg/ml for PD and 1.26 ± 0.002 pg/ml for RA) compared to control (1.13 ± 0.03 pg/ml; *p* < 0.05). *Clostridium perfringens* (100 µg and 400 µg) and *Bacteroides vulgatus* (400 µg) lysates and control were similar in respect to the secretion of IL-8 by PBMC and monocytes. Moreover, *Parabacteroides distasonis* lysates (at a dose of 100 µg and 400 µg) significantly increased IL-8 secretion by HT-29 cells (1.12 ± 0.02 pg/ml for 100 µg and 1.16 ± 0.002 pg/ml for 400 µg lysate) compared to *Bacteroides vulgatus* lysates (1.11 ± 0.001 pg/ml for 100 µg and 1.11 ± 0.001 pg/ml for 400 µg lysate; *p* < 0.05). In addition, *Clostridium perfringens*, *Ruminococcus albus* lysates (100 µg and 400 µg) and control were similar in respect to the secretion of IL-8 by HT-29 cells (Fig. 2).

## Discussion

The aim of the study was to determine the role and potential mechanism of action of intestinal microflora on secretion of cytokines and potential predisposition to asthma development. This study showed that *Parabacteroides distasonis* and *Ruminococcus albus* 400 µg lysate significantly increased secretion of IL-5 by PBMC compared to control (*p* < 0.05). In addition, *Bacteroides vulgatus*, *Clostridium perfrigens*, *Parabacteroides distasonis* and *Ruminococcus albus* 100 µg lysates significantly increased IL-8 secretion by PBMC and *Bacteroides vulgatus*, *Parabacteroides distasonis* and *Ruminococcus albus* 100 µg lysates by monocytes compared to control (*p* < 0.05). Moreover, *Parabacteroides distasonis* 100 µg and 400 µg lysates significantly increased the secretion of IL-8 by HT-29 cells compared to *Bacteroides vulgatus* 100 µg and 400 µg lysates (*p* < 0.05). Finally, *Clostridium perfrigens*, *Bacteroides vulgatus*, *Parabacteroides distasonis* and *Ruminococcus albus* 100 µg lysates significantly lowered IL-13 secretion by PBMC vs. control (*p* < 0.001).

Asthma is a chronic lung disease that leads to limited airway function due to inflammation, uncontrolled bronchial obstruction and accumulation of thick mucus in the airways^18,19^. Type 2 cytokines, such as: IL-4, IL-5 and IL-13, as well as IL-8 play a significant role in asthma development^20,21^. T helper 2 (Th2) lymphocytes and group 2 innate lymphoid cells (ILC2) activation, eosinophilic airway infiltration and secretion by such cells IL-4, IL-5 and IL-13, which lead to bronchial inflammation and remodeling are key mechanisms in the pathogenesis of asthma^22,23^. IL-4 has been involved in Th2 cells differentiation, as well as eosinophils influx (increased expression of VCAM-1) and chemotaxis (increased expression of eotaxin). Furthermore, IL-4 is responsible for lymphocytes B activation and immunoglobulin class switching (increased production of IgE) and mucus hypersecretion (induction of mucins gene expression)^22,24,25^. IL-5 plays a crucial role in differentiation, recruitment, activation and degranulation of eosinophils, which secrete many mediators, including: TGF-α/β, IL-2, IL-4, IL-5, IL-10, IL-12, IL-13, CCL5, CCL11, PAF and LCT4. Moreover, IL-5 is involved in tissue and airway remodeling^22,26,27^. IL-13 has an impact on increased secretion of IgE, increased production of NO (induction of iNOS expression), airway hyperresponsiveness (increased expression of FcεRI) and fibroblasts proliferation (activation of TGF-β1). In addition, IL-13 promotes construction and proliferation of airway smooth muscles and together with IL-4 increases mucus production and eosinophilic airway infiltration^21,22,24^. Finally, in some asthma patients, there is no eosinophilic inflammation. In such cases, airway inflammation is caused by activation, proliferation, and recruitment of neutrophils, for which IL-8 (secreted by macrophages, bronchial epithelial cells and smooth muscle cells) is responsible^20,28^.

Little is known about the role and potential mechanism of action of *Bacteroides vulgatus*, *Clostridium perfrigens*, *Parabacteroides distasonis* and *Ruminococcus albus* on the secretion of cytokines and chemokines and potential predisposition to asthma/atopic disorders development. To our knowledge, we are the first team to analyze such phenomenon for these four intestinal bacterial strains. *Bacteroides vulgatus* leaded to activation of NF-κB and NF-κB-p65 subunit nuclear translocation in HT-29/kb-seap-25, Caco-2 and LS174 T cells. Moreover, there was an increased expression of IL-6, IL-8, CXCL-10 and MCP-1^29^. Furthermore, stimulation of monocytes-derived macrophages (MDM), monocytes-derived dendritic cells (MoDC), bone marrow-derived macrophages (BMDM) with *Bacteroides vulgatus* LPS led to increased secretion of TNF-α (by MDM, MoDC and BMDM), IL-6 (by MDM and MoDC) and IL-10 (by MDM and MoDC) compared to untreated control. Furthermore, there was no change in IL-8 secretion in HEK293 cells stimulated by *Bacteroides vulgatus* LPS compared to control. Moreover, such cytokines were reduced in *Bacteroides vulgatus* LPS stimulated cells compared to *Escherichia coli* LPS stimulated cells^30^. *Parabacteroides distasonis* has been shown to exhibit anti-inflammatory properties in both in vivo and in vitro models. None of the tested strains caused overproduction of IL-8 in HT-29 cells^31^. *Clostridium perfringens* colonization of intestines was higher in infants with cow’s milk protein allergy^16^. It has been experimentally proven that the relative abundance of bacteria from the *Ruminococcaceae* family is lower in IgE-mediated eczema compared to the control group^32^.

A study published in 2017 indicated that bacteria from the *Lactobacillaceae* and *Mogibacteriaceae* families were negatively associated with asthma, while *Fusobacterium*, *Porphyromonas*, *Haemophilus* and *Neisseria* were positively correlated^33^. In 2021, scientists reported that the administration of both live and dead *F. prausnitzii* lowers the concentration of interleukins 4, 5, 13 and immunoglobulin G1, increases Tregs and improves dysbiosis^34^. Experiment conducted by Hessle et al. confirmed that Gram-positive bacteria triggered the release of TNF-α twice as much as Gram-negative bacteria. It should be noted that Gram-negative bacteria induced twice as many interleukins 6 and 8 than Gram-positive bacteria^35^. Atarashi et al. demonstrated that administration of mixed strains of *Clostridium* species (*p.o*.) to BALB/c mice stimulates allergen-induced expansion of Tregs in the colonic mucosa and reduces systemic IgE production^36^. Juan et al. noticed *Clostridium butyricum* CGMCC0313-1 diminished OVA-induced allergic airway inflammation in mice^37^.

This study had a several limitations. First, based on results of previous studies of gut microflora strains, we selected 100 µg and 400 µg lysates for our study. However, expanding the number of lysates tested in this study (e.g. to 5) would allow for a broader analysis of how increased amount of lysates of individual gut microflora strains affects changes in the secretion of the studied interleukins. Second, this study cannot recapitulate natural human in vivo exposure of these bacterial proteins to the human immune system which takes place in a complex 3D gut environment. Therefore, complex clinical studies with patients with asthma and/or atopy will be essential to precisely determine the effect of gut microbiota on the secretion of cytokines and chemokines associated with asthma/atopy. Finally, our study based on the analysis of four strains of gut microbiota on secretion of four interleukins by unstimulated commercially purchased cell lines. Moreover, we did not compare obtained results of normal cells to the cells isolated from asthma/atopic patients, to determine whether these strains would elicit different or the same response. Therefore, analysis of the effect of gut microbiota on the secretion of different mediators associated with asthma development in asthma/atopic and normal samples should be carried out in the future.

## Conclusions

This research confirmed that *Bacteroides vulgatus*,* Clostridium perfrigens*,* Parabacteroides distasonis* and *Ruminococcus albus* lysates modulate the secretion of IL-4, IL-5, IL-8 and IL-13 in human cells in vitro. The role of gut microbiota in asthma/atopic development is the subject of numerous studies. However, the studies conducted in this area are not unambiguous. Despite some limitations, this study provided novel information on the effect of gut microbiota on modulation of cytokines secretion by human cells and suggested future direction of the studies. For better understanding of the mechanisms of action of gut microflora and their impact on parameters important in asthma/atopy, complex studies which compare the asthma/atopic samples and control samples should be carried out in the future.

## Data Availability

The data used in the present study are available from the corresponding author upon reasonable request.
